# Blockchain and Random Subspace Learning-Based IDS for SDN-Enabled Industrial IoT Security

**DOI:** 10.3390/s19143119

**Published:** 2019-07-15

**Authors:** Abdelouahid Derhab, Mohamed Guerroumi, Abdu Gumaei, Leandros Maglaras, Mohamed Amine Ferrag, Mithun Mukherjee, Farrukh Aslam Khan

**Affiliations:** 1 Center of Excellence in Information Assurance (CoEIA), King Saud University, Riyadh 11451, Saudi Arabia; 2 Department of Electronics and Computer Science, USTHB University, Bab Ezzouar 16111, Algeria; 3 Department of Computer Science, King Saud University, Riyadh 11451, Saudi Arabia; 4 School of Computer Science and Informatics, De Montfort University, Leicester LE1 9BH, UK; 5 Department of Computer Science, Guelma University, Guelma 24000, Algeria; 6 Guangdong Provincial Key Lab of Petrochemical Equipment Fault Diagnosis, Guangdong University of Petrochemical Technology, Maoming 525000, China

**Keywords:** industrial IoT, industrial control system, SCADA, distributed control system, blockchain, software-defined network, random subspace learning, intrusion detection system, security

## Abstract

The industrial control systems are facing an increasing number of sophisticated cyber attacks that can have very dangerous consequences on humans and their environments. In order to deal with these issues, novel technologies and approaches should be adopted. In this paper, we focus on the security of commands in industrial IoT against forged commands and misrouting of commands. To this end, we propose a security architecture that integrates the Blockchain and the Software-defined network (SDN) technologies. The proposed security architecture is composed of: (a) an intrusion detection system, namely RSL-KNN, which combines the Random Subspace Learning (RSL) and K-Nearest Neighbor (KNN) to defend against the forged commands, which target the industrial control process, and (b) a Blockchain-based Integrity Checking System (BICS), which can prevent the misrouting attack, which tampers with the OpenFlow rules of the SDN-enabled industrial IoT systems. We test the proposed security solution on an Industrial Control System Cyber attack Dataset and on an experimental platform combining software-defined networking and blockchain technologies. The evaluation results demonstrate the effectiveness and efficiency of the proposed security solution.

## 1. Introduction

With the industrial revolution, we have witnessed rapid changes in factory automation, transportation security, and surveillance in large-scale industries. To this end, Industrial IoT (IIoT) [1] has drawn a significant interest by incorporating dense wireless devices such as Radio-Frequency IDentification (RFID) tags [2] for machine identification, sensors for large-scale equipment monitoring and fault diagnosis, production, manufacturing, asset monitoring and many applications for power plant, water supplies, oil, and gas refineries.

Industrial control systems (ICS) are used to describe different systems such as Supervisory Control and Data Acquisition (SCADA) and Distributed Control system (DCS). SCADA collects and analyzes data from substations in real-time. Each substation contains control devices, such as Programmable Logic Controller (PLC), Remote Terminal Unit (RTU), and Intelligent Electronic Device (IED), which manages field devices, such as sensors, actuators, and meters. The collected field information is sent to a central facility, which consists of (1) SCADA server to process the information, and (2) Human Machine Interface (HMI) for information displaying. DCS, on the other hand, focuses on the automatic control of the industrial infrastructure. Other applications like: data historian, Manufacturing Execution Systems (MES), and Enterprise Resource Planning (ERP).

**Figure 1:**
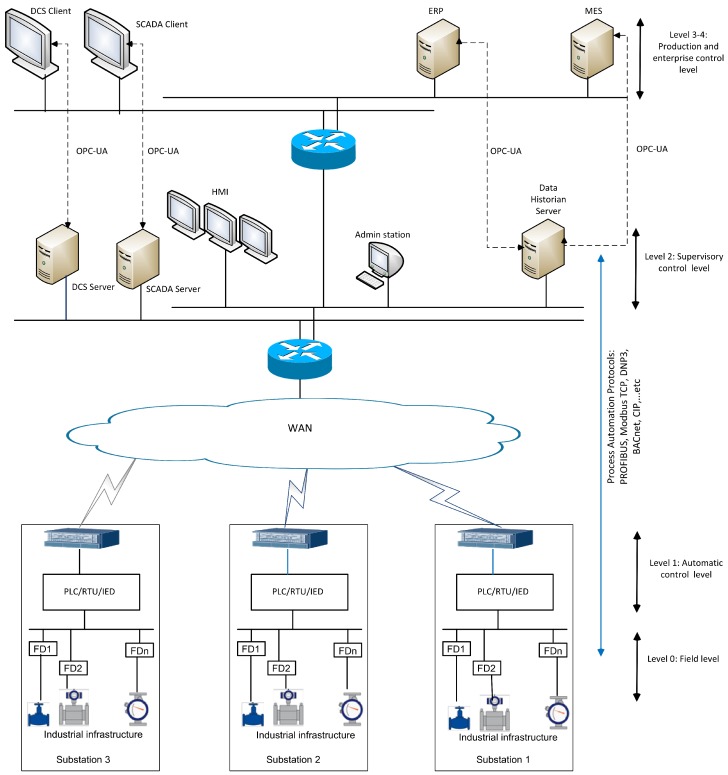
Typical architecture of industrial control systems (ICS) over Wide-Area Network (WAN).

Wide-Area Network (WAN) connections, such as Broadband Internet, Fourth generation (4G) communication, Long Term Evolution (LTE), or Multiprotocol Label Switching (MPLS) allow ICS to extend their networks to large distances in order to connect remote substations. Industrial communication protocols like: Modbus TCP, DNP3, and OPC-UA are used to exchange data between the different components of the industrial control system. Figure 1 shows the typical architecture of ICS over WAN. Industrial control systems are used in different critical infrastructures where IIoT can play a major role, such as in power plants, water supplies, oil, gas, and renewable energy facilities.

In fact, with the autonomous exchange of data among devices and a server, or in a device-to-device manner, either directly or over a network, will benefit the industrial control system to control and monitor the industrial process locally or at the remote location. The integration of IoT solutions with ICS, named also *fourth Generation ICS* [3,4], allows collecting and analyzing a large data set over the whole industrial area. By this way, this integration is foreseen as a viable solution towards smart and efficient data gathering and aggregation frameworks for the entire automation industry [5].

Industrial Control Systems (ICS) are becoming primary targets of cyber attacks due to their increased interconnection with other corporate networks. Their exposure to private and public networks has increased the risk of such attacks targeting ICS in recent years [6]. These attacks cause a variety of damages and drastic consequences to humans and their environment. For instance, a power blackout in Ukraine’s capital Kiev happened because a SCADA system, which was linked to the 330 kilowatt substation, was influenced by external sources outside normal parameters [7]. In addition, as ICS deploys a large number of network devices like routers and switches, they bring other security issues. As each device represents a possible entry point to the attacker, the more devices we have, the more risks ICS is exposed to. Besides, network devices require continuous management and configuration, which is costly and time-consuming. To deal with this issue, software-defined networking (SDN) [8,9] technology was proposed to facilitate software and hardware updates on the network devices. This is achieved by moving the control of lookup tables stored in the network devices to a central location that allows easy control and management. In this way, the risk of compromising the network devices could significantly be reduced. Software-defined wide-area network (SD-WAN) [10] is a specific application of the SDN technology that is applied to WAN connections. Similar to SDN, SD-WAN also decouples the networking hardware from its control mechanism. However, SD-WAN focuses more on cost savings by reducing the deployment and operational costs. Gartner [11] predicted in 2015 that 30% of enterprises would deploy SD-WAN technology in their branches by the end of 2019.

In this paper, we propose a security architecture for the industrial control system, which is integrated with the SD-WAN technology. The architecture considers the attacks that target the ICS commands, and negatively affect the correct functionality of the ICS. The attacks are classified into two types: (a) *forged ICS command* that target the industrial control process, and (b) *misrouting of commands* that is brought to the surface due to the adoption of the software-defined technology, e.g., an adversary that injects fraudulent flow rules, which prevent correct routing of ICS commands and information. Thus, the proposed security architecture requires two main complementary components: (a) an intrusion detection system to defend against the forged commands, and (b) an SD-WAN-based security solution, which prevents the misrouting of commands and information through tampering of the flow rules. The main contributions of the paper are the following:

- We propose an SD-WAN architecture for industrial control systems.
- We define the attack model that can target the proposed architecture. The attack model comprises: forged command attacks that target the industrial control process, and SDN-related attacks that misroute commands and information.
- We propose a security solution for the proposed SD-WAN architecture that includes two complementary components:
  - An intrusion detection system (IDS), named RSL-KNN, against forged command attacks that target the industrial control process, which leverages the random subspace learning approach and K-Nearest Neighbor (KNN) classifier to outperform conventional machine learning classifiers.
  - A Blockchain-based Integrity Checking System (BICS), which can defend against the misrouting attack, by detecting in a short time any tampering with the OpenFlow rules and preventing the execution of the rules. Differently from [12,13,14] that detects this attack by analyzing the flow rules, our system is lightweight in the sense that it only compares the traffic flow rules, which are originated from the vSwitch, with the one sent by the SDN controller.
- We evaluate the effectiveness and efficiency of the proposed security solution. By applying the random subspace learning-based IDS on the Industrial Control System Cyber attack Dataset [15,16], promising accuracy results are achieved. On the other hand, a blockchain-based integrity checking system is able to detect all attacks against the flow rules at a very low detection time.

The remainder of the paper is organized as follows: Section 2 provides the related work. In Section 3, we present the SD-WAN architecture for the industrial control system along with the attack model. In Section 4, we describe the main components of the security solution for the SDN-based ICS. The implementation and evaluation of the proposed security solution are presented in Section 5 and Section 6, respectively. Finally, Section 7 concludes the paper.

## 2. Related Work

### 2.1. Intrusion Detection Systems for ICS

In order to protect the industrial control systems, several recommendations and good practices can be followed [17]. One important security component that can be used in order to protect these assets against new threats is an IDS. Especially, for identifying anomalous or unexpected behavior in ICS systems, anomaly detection systems have shown great potential [18]. The IDS must be combined with active network monitoring mechanisms for collecting the necessary data along with traditional defense mechanisms like firewalls and antiviruses. Recently, authors in [19] proposed a new taxonomy of ICS IDS by taking into account the characteristics that industrial systems have. Moving away from standard taxonomy like rule-based, misuse detection and mixed systems, the authors divided them into three new categories, i.e, protocol analysis-based, traffic mining-based, and control process analysis-based detection systems. There exist a number of state-of-the-art machine learning-based big data processing technologies for anomaly detection [20]. Although the IDSs that fall into the first two categories manage to detect standard cyber attacks by analyzing the used protocols or the traffic data that is exchanged between different entities of the system, they fail at detecting the so-called semantic attacks. Semantic attacks exploit the knowledge extracted from the normal operation of an ICS about the control systems that are in place or the physical processes that take place, taking into account the close association between ICS and physical systems. Ignoring semantics provides the attackers the chance to gain control of some industrial processes and launch attacks that may tamper the normal operation of some physical devices or change the operating rules on field devices. These attacks pass unattended from traditional IDSs since they do not violate the specifications of the protocols or create any abnormal network traffic in the system. Narayanan et Bobba [21] moved one step further by proposing an application-level anomaly detection framework that detects attacks aiming to modify the products that were manufactured from the industry that uses the ICS.

Many researchers came up in recent years with proposals that took advantage of machine learning models for developing IDSs to protect the control process. Caselli et al. [22] discussed sequence attack scenarios within an industrial control system and developed a sequence-aware intrusion detection system. Khalili et al. [23] proposed a State-based Intrusion Detection System (SIDS) suitable for cyber physical systems. Contrary to other industrial IDSs that consider anomalous states as indications of the cyber attacks, SIDS manages to detect three types of attacks: anomalous states, anomalous transitions between the normal states, and anomalous time-intervals between the normal transitions. Zhang et al. [24] proposed a detection system for attacks against cyber physical systems. The proposed system studied four classical classification models, including k-nearest neighbor (KNN), decision tree, bootstrap aggregating (Bagging), and random forest. To strengthen early attack detection, the proposed system uses an auto-associative kernel regression model. Abokifa et al. [20] developed an algorithm for the detection of cyber-physical attacks and adjusted for smart water distribution systems. Ghaeini et al. [25] proposed a framework for state aware anomaly detection in industrial control systems that is proven to provide lower exposure time while offering better detection in terms of false alarm rate. Trying to cope with data injection attacks in ICS, Wang et al. [26] proposed a new method that uses a Long Short Term Memory Recurrent Neural Network (LSTM-RNN) as a temporal sequences predictor. Li et al. [27] proposed a SCADA firewall model, called SCADAWall, for SCADA security. The SCADAWall model is powered by a comprehensive packet inspection (CPI) technology. To extend capabilities to proprietary industrial protocol protection, the SCADAWall model uses a proprietary industrial protocols extension algorithm (PIPEA). Based on the out-of-sequence detection algorithm, the SCADAWall model can detect abnormality within industrial operations. Finally, several recent review articles discuss issues related to IDS systems for ICS and SCADA [28,29], all of them showcasing the need for novel sophisticated solutions.

### 2.2. Risk and Threat Modelling for ICS

Falco et al. [30] presented a study on the Industrial Internet of Things (IIoT) cybersecurity risk modeling for SCADA systems. Specifically, the study demonstrated that certain risk metrics are stronger indicators than others in evaluating the likelihood of exploits for SCADA systems. Wood et al. [31] after identifying current risks with SCADA and ICS systems proposed a two layered security architectural pattern to address them. On the other hand, Cook et al. [32] conclude that the parameters for defining risk are too many (consequences C, events A, background information K, measure of uncertainty Q, threat T, vulnerability V) and there is no proven unified risk model exists for ICS that incorporates all of them yet, leaving this important field open for future research.

Nourian and Madnick [33] presented a study about how the vulnerabilities were exploited by Stuxnet, which is an attack designed to interrupt the Iranian nuclear program. Based on prior research on system safety, the study uses a system theoretic approach in order to analyze the threats exploited by Stuxnet. This approach can identify cyber threats towards CPSs at the design level and provides practical recommendations for more secure SCADA and automation systems. Nasr and Varjani [34] proposed an alarm-and-trust based access management system named ATAMS, to reinforce the security of the SCADA system against the deontological threats. The ATAMS system can reduce the deontological threats based on the two levels, including, (i) integrity level of the substations and (ii) the operator trust value.

### 2.3. Blockchain for IoT and SCADA Security

The blockchain technology can be effectively applied in almost all domains of the IoT, especially when the IoT applications demand a decentralized security framework, as presented in [35]. Košt’ál et al. [36] proposed an improved architecture for management and monitoring of IoT devices using a private blockchain. Agyekum et al. [37] proposed a proxy re-encryption scheme that incorporates an inner-product encryption scheme for IoT environments. In order to improve the routing security and efficiency for the internet of sensors, Yang et al. [38] proposed a trusted routing scheme using blockchain and reinforcement learning.

The SCADA can be more intelligent and smart using the IoT networks. However, distributed blockchain-based methods have been proposed recently to detect and defend against cyber-attacks for modern power systems. Liang et al. [39] proposed a blockchain-based framework based on three phases, namely, data transmission, verification, and storage. The data transmission phase uses two keys, including: (1) the public key available in the meter-node network, and (2) the private key containing the node’s private information. The verification phase uses an address-based distributed voting mechanism in order to verify the data integrity. The block generation phase uses two strategies as solution methods, including: (1) generating block by a fixed time, and (2) generating block by a fixed size. Liang et al.’s framework can be considered as a promising solution but the privacy and anonymity are not considered, which can compromise the energy trading infrastructure (e.g., using attack tree). To trade energy in a peer-to-peer network without a central price signal, Aitzhan and Svetinovic [40] proposed a token-based energy trading system called PriWatt, for the smart grid using distributed smart contracts. Specifically, PriWatt system uses signing transactions and multi signatures. To validate the authenticity of a transaction, PriWatt system uses ECDSA asymmetric cryptography. Therefore, a transaction can be considered valid only when multiple independent parties sign this transaction. Remarkably, PriWatt system provides certain levels of privacy and security as well as combats double-spending attacks.

### 2.4. SDN-Based SCADA

Several works [41,42] studied the integration of SDN with SCADA systems to facilitate better management and configuration of the network devices. Due to the centralized controller with a comprehensive view, the network operation can be better optimized compared to the traditional management in SCADA systems. Other works focused on designing SDN-based resilient solution for smart grids [43,44,45,46,47]. This is achieved by deploying redundant communications. In case of failures, the communication and services are quickly restored. A Network-based Intrusion Detection System (NIDS) for SDN-based SCADA systems is proposed in [48] using One-Class Classification (OCC) algorithm on the gathered statistics from network devices. Basically, the SDN features were leveraged to frequently modify the multipath routing mechanism between the SCADA devices. One interesting advantage is that the IDS can detect possible malicious traffic with unknown attack signatures. Most recently, a framework with multiple SDN controllers and security controllers is suggested in [49] for SCADA. The local IDS in the substation collects the measurement data periodically and monitors the control-commands. Furthermore, the global IDS that resides in the control center collects the measurement data from the substations and detects any abnormal behavior of the control-commands issued by the SDN controller and SCADA. In addition, a light-weight identity based cryptography is suggested to protect the network from outsider attacks.

### 2.5. Fraudulent Rule Detection in SDN

In the literature, there are some surveys [50,51] that describe the attacks that can target the SDN along with their corresponding security methods. Some methods such as FortNOX [13], FlowChecker [12], and VeriFlow [14] are designed to detect fraudulent rules. FortNOX [13] developed an analysis algorithm to detect rule conflicts. A rule conflict occurs when a new OpenFlow rule is in contradiction with the existing rules. FlowChecker [13] analyzes all switch configurations using a binary decision and model checking to detect misconfigurations within the flow tables. Veriflow [14] checks for any violation of network invariants (e.g., availability of a path towards the destination, absence of routing loops) when a new rule is inserted, deleted, or updated. In a multi-controller architecture, the detection of fraudulent rules and malicious controller in [52,53] is achieved by replicating each rule setup request to multiple controllers simultaneously in order to check for rule consistency, which incurs a high overhead. In order to deal with the issue, requests are randomly replicated in [54].

### 2.6. Comparison with Related Work

To the best of our knowledge, our work is the first that combines the SDN and blockchain technologies into one architecturally secure design for industrial control systems. In addition, it differs from the related work in the following points:

1. Random subspace learning has never been investigated as an IDS approach for industrial control systems to distinguish between normal events and cyber attacks.
2. We show that combining random subspace learning and K-Nearest Neighbors improves the IDS accuracy compared to the basic machine learning classifiers, such as SVM, decision tree, random forests, etc.
3. Differently from FortNOX [13], FlowChecker [12], and Veriflow [14] that detect fraudulent rules based on a heavyweight analysis process, the proposed BICS architecture is lightweight in the sense that it only leverages the blockchain technology to compare the traffic flow rules, which are originated from the vSwitch with the one sent by the SDN controller.
4. BICS provides a scalable solution under multi-controller environment, as there is no need to involve multiple controllers to check the rule correctness.

## 3. SD-WAN Architecture for Industrial Control Systems

We propose an SD-WAN architectural design for ICS that enables network virtualization by migrating the control layer to the cloud, which helps to allow a centralized management. As legacy WANs can be costly and complex, SD-WAN architecture reduces the network cost by offering zero-touch deployment, i.e., there is no need to configure the network device by plugging it in. Instead, the device is configured from the SDN controller. In terms of security, the architecture can provide a unified security policy across the network.

**Figure 2:**
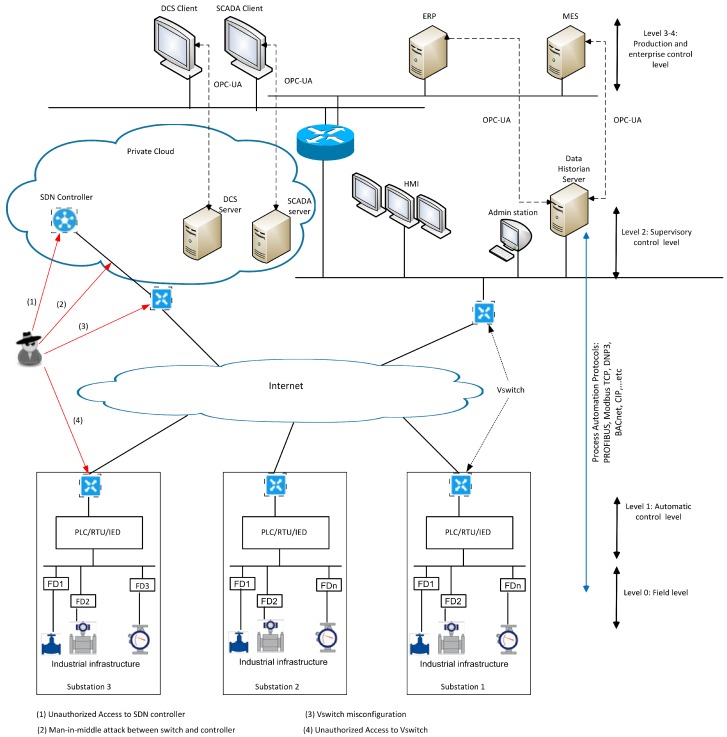
Software-defined networking (SDN)-based ICS architecture.

As shown in Figure 2, the proposed architecture is composed of the following components:

- *Private cloud*: It hosts all the components that offer a centralized control for ICS as virtual machines, such as SCADA server, DCS server, and SDN controller.
- *IP network*: Instead of using a dedicated WAN for ICS, we can use the public Internet connection between the SDN and the different substations. All devices are authenticated and end-to-end encryption is established across the network.
- *SDN controller*: It is an application that manages flow control by using protocols such as OpenFlow [55] that tells switches where to send data packets. The OpenFlow protocol is a southbound interface between the controller and the forwarding elements such as switches. The northbound interface considers the communication between the controller and the applications.
- *Virtual Switch*: It is an application that interconnects multiple virtual machines of the same or different hypervisors. Moreover, it also interconnects these virtual machines with other physical switches.

Based on the above architecture, we present the attack model that targets the security of ICS commands, and could adversely affect the correct functionality of ICS. In the following, we consider two types of attacks:

- *Forged command:* Attacks that issue forged commands to intelligent electronic devices, which trigger the execution of undesired operations, such as blackout.
- *Misrouting of commands:* Attacks that prevent the correct routing of commands and other information between the SCADA server, DCS server, and the different devices of ICS. This attack can be achieved by modifying the flow rules.

Specifically, the above mentioned attacks can be further classified as follows:

- *Forged command:* This type of attack considers the injection of fraudulent flow rules in the flow table. Under this type, we can find the following attacks:
  - *Vswitch misconfiguration*: Similar to the physical switch, the virtual switch might allow devices impersonating another device so that it can receive network frames intended for that device. In addition, the virtual switch might be configured to allow a device receiving frames targeted for other devices. In this way, an attacker can masquerade as the SDN controller and insert fraudulent rules in the flow tables of other vSwitches.
  - *Unauthorized Access to SDN controller*: When an attacker impersonates an SDN controller, it can gain access to the network resources and control all the network operations, including injecting fraudulent rules in the flow tables.
  - *Man-in-the-middle attack between switch and controller*: An attacker, by spoofing the identities of the two nodes, can secretly intercept and modify the communication between them. This attack can be achieved through different mechanisms, such as vSwitch misconfiguration or port mirroring. In this way, it can send fake flow rules to the vSwitches.
- *Misrouting of commands:* This type attacks the control process of the ICS by performing *Unauthorized Access to vSwitch*. Due to the vSwitch misconfiguration, the attacker can gain access to the vSwitch of the substation and issue fake commands to the different control devices.

We consider that the security of industrial communication protocols like OPC-UA, DNP3, and Modbus is not within the scope of this work, as they have already been analyzed [56,57,58,59].

## 4. Security Architecture Description

**Figure 3:**
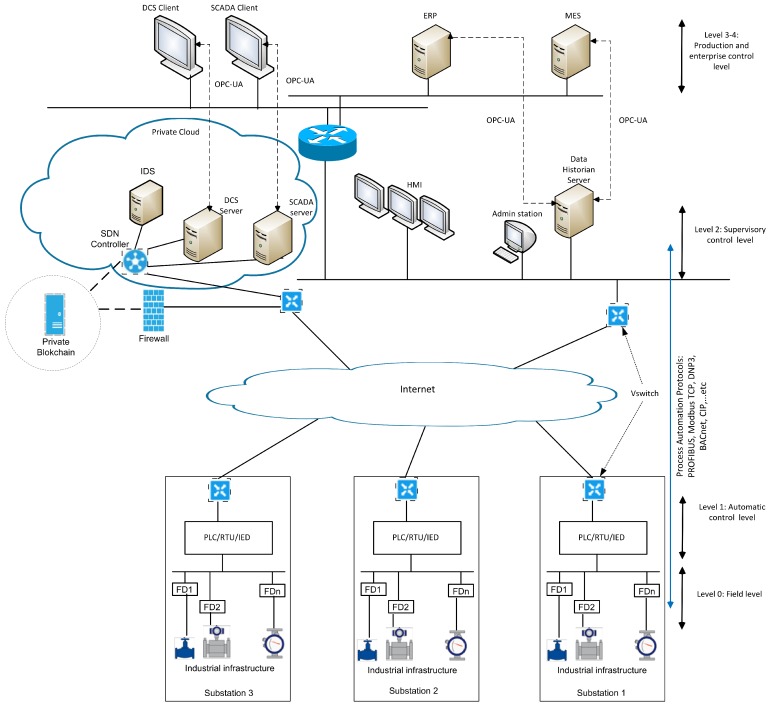
Security architecture for SDN-based ICS.

In this section, we propose two security components, as shown in Figure 3, to address the attack model defined in Section 3, which are:

- Intrusion detection system (IDS) to identify malicious command issued to the control devices. In this work, we propose an IDS using the Random Subspace learning (RSL). Later, in Section 6, we show that RSL-KNN, which is the combination of RSL and KNN classifiers, gives better results than the conventional machine learning classifiers.
- Blockchain-based integrity checking system (BICS), which aims to detect any injection of fraudulent flow rules in the vSwitches.

### 4.1. Random Subspace Learning-Based IDS

The concept of random subspace learning is proposed by Barandiaran [60]. The Random Subspace Learning (RSL) method is an ensemble learning technique, which is also called features bagging or attributes bagging [61]. It is used to improve prediction and classification tasks as: (1) it employs ensemble construction of base classifiers instead of a single classifier, and (2) it takes random subsets of features instead of the entire set of features. In this way, the correlation between features among classifiers is reduced. This method has proved its success in a lot of prediction and classification problems [62,63,64,65].

**Figure 4:**
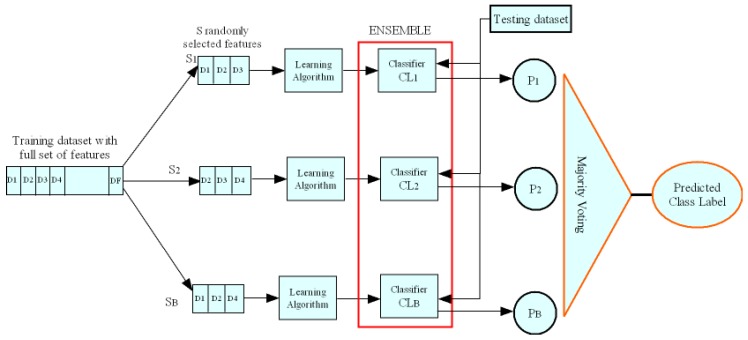
Random Subspace Learning process: training and testing.

The random subspace learning process is illustrated in Figure 4, and consists of two phases: training and testing.

In the training phase, we randomly select *S* features from a set of *F* features such that S≤F. The selected features are fed to a machine learning algorithm to generate a classifier/learner. This operation is repeated *B* times, and at each time *S* features are picked at random with replacement to generate a different classifier.

In the testing phase, the outputs from all distinct learners are combined by majority voting to obtain the final prediction or classification result. The main advantage is that combining classifiers improves the accuracy, especially if the classifiers are independent, or not correlated with each other through features. In other words, the classifiers are fed with different sets of features from each other, which reduces the correlation between features among classifiers.

More specifically, we assume that the RSL model contains a number of individual classifiers, which are built from *S* subspaces of features defined as ${\left\{C_{i}\left(.\right)\right\}}_{i=1,\cdots L}$. The number of labels returned by the individual classifiers will be given as ${\left\{\bar{y_{i}}\right\}}_{i=1,\cdots L}$, where the returned labels belong to the set of labels (*Y*) in the training dataset.

For unseen instances $x_{\left(k=1,\cdots X\right)}$ of *F* features, each classifier will classify them based on its features subspace $S_{j=1,\cdots f}\in F$ separately. Then, the outputs from separate classifiers are represented as:

(1) $$\bar{y_{i}}=C_{i}\left(x_{k},S_{j=1,\cdots f}\right).$$

Finally, all outputs from separate classifiers are combined using the majority voting Algorithm [63] to obtain the final classification label *y* as in the following equation:

(2) $$y=\arg\max_{\bar{y_{i}}}\sum_{i=1}^{L}\bar{y_{i}}\;\in Y.$$

More formally, Algorithm 1 shows the steps to generate the ensemble of random subspace classifiers, and the ones to compute the predicted labels of unseen instances. Let $T_{N}^{F}$ denote the original training dataset of *F* features and *N* instances, $T_{N}^{S}$ denote the partial training dataset instances of only *S* features, which are randomly selected from the original training dataset, $Z_{M}^{S}$ represents the testing dataset of *M* instances with the same selected features *S* as the ones selected in the training phase, ML denotes the machine learning algorithm. In the training phase, we take ML and $T_{N}^{S}$ as input *B* times to generate a classifier $CL_{b}$, 1≤b≤B. In the testing phase, we compute $P_{b}^{M}$, which represents the classification labels of *M* unseen instances using the base classifier $CL_{b}$. Then, we compute $P_{l}^{M}$, which is the final classification labels of *M* instances after majority voting of the base classifiers.

As will be seen in Section 5, RSL-KNN classifier is obtained by combining random subspace learning and KNN algorithm. In other words, we get RSL-KNN by setting ML (resp., replacing the Learning Algorithm component) to KNN (resp., with KNN) in Algorithm 1 (resp., Figure 4).

**Algorithm 1** Random Subspace learning classifier.
**Input:**$T_{N}^{S}$, $Z_{M}^{S}$, *B*, ML
**Output:** $Y_{l}^{M}$

1:**for**b=1 to *B***do**                                ▹ Training phase2:  Set the dimension S≤F;3:  Select randomly *s* features from $T_{N}^{F}$ to derive $T_{N}^{S}$;4:  $CL_{b}=Build\_RSL\left(T_{N}^{S},ML\right)$▹ Build Random subspace learning classifier based on dataset $T_{N}^{S}$ and machine learning algorithm ML;5:
**end for**
6:**for**b=1 to *B***do**                                ▹ Testing phase7:  $P_{b}^{M}⟵CL_{b}\left(Z_{M}^{S}\right)$            ▹ Classify $Z_{M}^{S}$ instances using the built model;8:
**end for**
9:$P_{l}^{M}=MajorityVote\left({\left\{P_{b}^{M}\right\}}_{b=1}^{B}\right)$   ▹ Find predicted class labels $Y^{M}$ using Majority voting of base classifier models;10:**return**$P_{l}^{M}$;


### 4.2. Blockchain-Based Integrity Checking System

Before describing the security solution, we make the following assumptions:

- We assume that the SD-WAN ICS is not compromised (i.e., free from malicious code before the installation of the Blockchain-based integrity checking system. Otherwise, forged rules can be considered as legitimate.
- The Blockchain-based integrity checking system only focuses on southbound communication. We assume that the northbound communication between SCADA server, DCS server, and IDS from one side and the SDN controller from the other side, is secure.
- We assume that the SDN controller is located in a private cloud, and only accessible from a single host through an authentication and access control mechanism.

**Figure 5:**
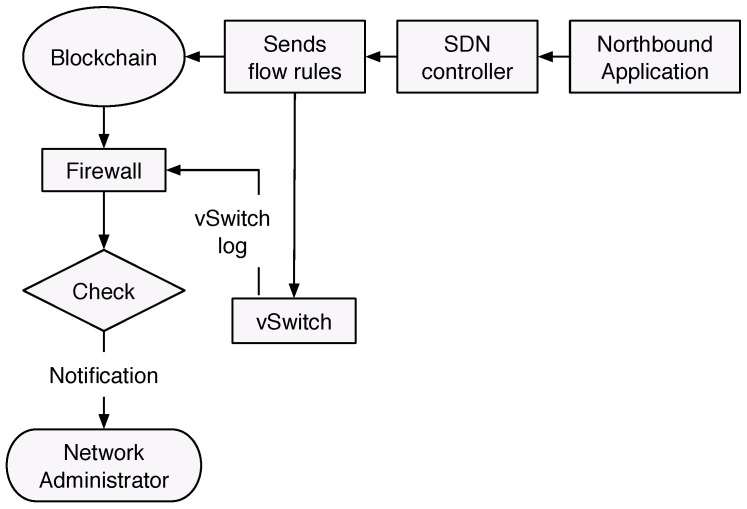
Flowchart execution of blockchain-based integrity checking system (BICS).

The Blockchain [35] is the key element in the design of our integrity checking systems. The basic idea is to provide a solution where all flow rules that are generated from the controller are stored in a verifiable and immutable database. The blockchain is a sequence of blocks, which are linked together by their hash values. In the blockchain network, each user has two keys: one private key to sign the blockchain transaction and one public key that represents its unique address. The user signs a transaction using its private key and broadcasts it to its peers in the network for validation. After validating the broadcast block, which contains the transaction, it is appended to the blockchain. Once recorded, the data in any given block cannot be changed without alteration of all subsequent blocks. In addition, the data exists in multiple hosts at once, so any changes would be rejected by the peer’s hosts. In this work, we proposed a private (or permissioned) blockchain. Differently from the public blockchains, the private ones determine who is allowed to participate in the network, and defined actions and permissions are assigned to identifiable participants. Hence, consensus mechanisms such as Proof of Work are not required. Our blockchain is composed of only two nodes: SDN controller, and firewall. The SDN controller creates blocks and shares it with the firewall via the blockchain. The first node has all the permissions, i.e., read, write, and send, whereas the firewall can only read and receive. As shown in Figure 5, the blockchain-based integrity checking system is carried in the following sequences:

- Upon receiving a request from the Northbound application, the SDN controller is designed to send the corresponding flow rules to the vSwitches. In our design, the SDN controller is also a member of a blockchain. It hashes the flow rules and puts them in a block that is distributed to the other nodes of the blockchain. The SDN controller is the only node in the blockchain, which has the right to create blocks, whereas the rest of the nodes can only read the blockchain.
- When the flow rules reach the vSwitch node, the latter updates its flow table and saves the rules in the log file.
- The Firewall collects the vSwitch logs and accesses the BlockChain to obtain the flow rules sent by the controller.
- If the firewall finds that the two rules, from vSwitch and blockchain, are not similar, it notifies the Administrator to take the appropriate countermeasures to fix this mismatching.

## 5. Implementation

### 5.1. Random Subspace Learning-Based IDS

**Figure 6:**
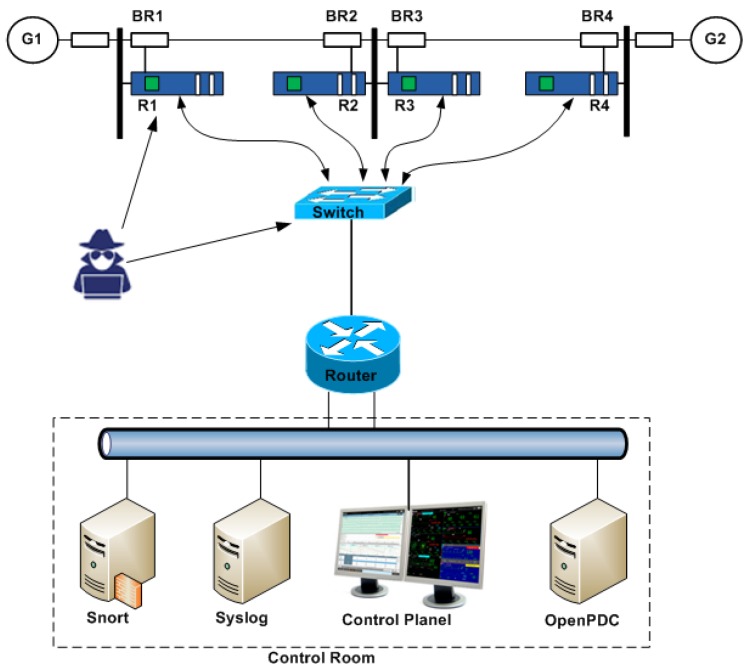
Case study: industrial control power system ([16]).

In this section, we evaluate the performance of the proposed IDS using a real case study scenario that is implemented in [16], and using the Power System Dataset, which is a part of the Industrial Control System Cyber Attack Dataset [15]. Figure 6 shows the industrial control power system architecture. It is composed of the following components:

- Two power generators: G1 and G2.
- Four breakers from BR1 to BR4.
- Two transmission lines: L1 between BR1 and BR2, and L2 between BR3 and BR4.
- R1 through R4 are intelligent electronic devices (IEDs) to switch the breakers on or off. The IEDs send information to the control room through a substation switch and a router.

As explained in [16], there are four synchrophasors, each of which measures 29 features, which give in total 116 phasor measurement unit (PMU) measurements. There are also additional 12 features from control panel logs, Snort logs and relay logs. Thus, 128 features are used in this case study scenario. Examples of some features, which are extracted from each PMU, are as follows.

- *PA1:VH-PA3:VH:* This feature represents phase A-C voltage angle.
- *DF:* This feature represents frequency delta ($\frac{df}{dt}$) for relays.

The list of 128 features along with their descriptions are given in [15]. The dataset [15] considers the following two normal events and three attack events. The normal events are as follows:

- *Short-circuit fault:* It represents a short in a power-line and can occur at different locations along the line.
- *Line maintenance:* Power system operators occasionally must take a transmission line out of service to allow maintenance.

The dataset also considers the following attack events:

- *Remote tripping command injection:* This attack sends a command to a relay and causes a breaker to open.
- *Relay setting change:* Relays are controlled via configurable settings. Certain settings exist to disable relay operations. This class of attacks alters relay settings to disable relay operation such that the relay will not trip for valid commands or faults.
- *Data Injection:* This attack aims to imitate a valid fault by altering system measurements followed by sending an illicit trip command from a compromised computer to relays at the ends of the transmission line. This attack aims to blind the operator and causes a blackout.

The dataset is composed of 15 sub-datasets, as shown in Table 1. The events in SCADA systems are used in the following two main classification tasks:

- Classification of multi-class events: This classification task contains 37 scenarios of events, and includes normal event, natural event, and attack events with their own class labels.
- Classification of binary class events: This task also contains 37 event scenarios, which are divided into nine normal events and 28 attack events. All the 15 sub-datasets consist of thousands of distinct event types and are sampled at 1% in a random manner. Therefore, each sub-dataset contains 3711 attack instances, 294 samples of *no event* instances, and 1221 *natural events* instances. Table 1 summarizes the distribution of instances in the 15 SCADA sub-datasets.

The RSL method is also implemented using Weka tool [66]. Table 2 summarizes the parameters that are considered in the implementation. A 10-fold cross-validation strategy is also adopted to apply the proposed method on the 15 SCADA sub-datasets.

### 5.2. Blockchain-Based Integrity Checking System

**Figure 7:**
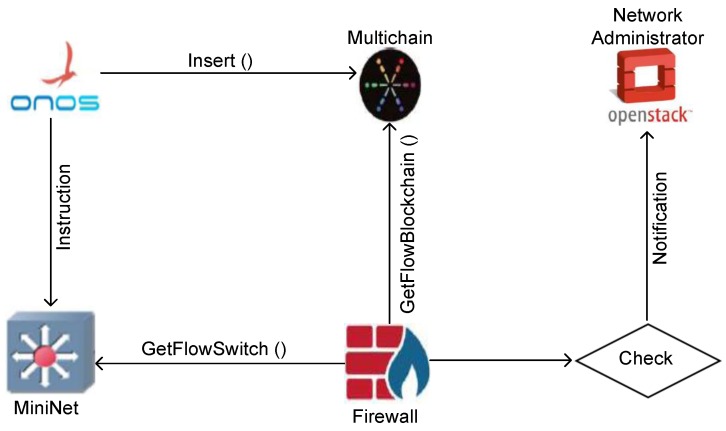
Implementation components of BICS.

The Blockchain-based integrity checking system is implemented, as shown in Figure 7, using the following components:

- *Private cloud*: We use Openstack [67] to implement the private cloud.
- *BlockChain*: We use Multichain [68], which is derived from Bitcoin Core [69], to implement a private blockchain. It uses JSON [70] to create blocks. The role of this blockchain is to save all the operations transmitted from the SDN controller to the different switches. Multichain ensures the following properties:
  - The activity of the blockchain is only visible to the chosen participants.
  - It provides read and write privileges on the transactions
- *SDN controller*: We use ONOS [71] to implement SDN controller. ONOS is an SDN that provides the control plane of the network. It manages its components such as switches and routers, and links. It runs the software that provides communication services to end-users and neighboring networks.
- *Mininet*: Mininet [72] is a network emulator, which creates a network of virtual hosts, switches, controllers, and links. It allows creating an SDN prototype to simulate a network topology using switches supporting OpenFlow.

As shown in Figure 7, the Insert() program captures the traffic sent from the ONOS controller to the vSwiches in order to get the flow rules of the ONOS controller and save them on the blockchain. In order to access the blockchain, write permissions to create blocks on the blockchain are assigned to this program. Each created block contains the following information:

- *PUBLISHER*: the SDN controller identifier.
- *Data*: The flow rule, and consists of the following records:
  - *ID*: The identifier of the rule.
  - *TABLEID*: The flow table identifier.
  - *DEVICEID*: The Vswitch identifier.
  - *TYPE*: The rule type: input/output.
  - *OUTPORT*: The output port number.
  - *INPORT*: The input port number.
  - *PRIORITY*: The priority of the rule.
  - *MACSRC*: The source MAC address of flow.
  - *MACDST*: The destination MAC address of flow.
- *BLOCKTIME*: The creation time of the block.
- *BID*: The block ID.

## 6. Performance Evaluation

In this section, we evaluate the performance of two components of our solution: Random Subspace Learning IDS, and Blockchain-based integrity checking system (BICS).

### 6.1. Random Subspace Learning IDS

In the following, we present the performance results of the Random Subspace Learning IDS with respect to effectiveness and efficiency.

#### 6.1.1. Effectiveness Evaluation

In this section, we use a set of baseline machine learning classifiers to test their ability to detect SCADA attacks. The used baseline classifiers are as follows: Linear Support Vector Machine (LSVM), Bayes Network (BN), Naive Bayes with kernel estimator (NB-K), K-Nearest Neighbor (KNN), AdaBoostM1, Bagging, Decision Tree (DT), and Random Forests (RF). All these classifiers are applied on the 15 SCADA datasets and implemented using the open-source tool of machine learning, namely Weka [66]. In the implementation, we fix the number of iterations for ensemble classifiers to be 25, the block size to be 100, and the bag size to be 50. Other settings remain as default. A 10-fold cross validation strategy is used in the testing. This strategy randomly partitions the datasets into 10 sets of instances and selects one set for testing and the other nine sets for training. We repeat this strategy 10 times and take the average to summarize the results for each of the used 15 sub-datasets.

The evaluation results are presented under the following metrics:

- Accuracy $=\frac{TP+TN}{TP+TN+FP+FN}$
- False Positive Rate $=\frac{FP}{FP+TN}$

where TP, TN, FP, and FN denote true positive, true negative, false positive, and false negative, respectively.

Table 3 and Table 4 show the accuracy results of the above-mentioned machine learning classifiers under binary classification (natural and attack) and multi-class classification (natural and different types of attacks), respectively.

Motivated by the accuracy results of intrusion detection for KNN classifier compared to other classifiers, we propose an effective method that combines the random subspace method with KNN classifier, named Random Subspace Learning-based K-Nearest Neighbor (RSL-KNN) method. The basic idea behind RSL-KNN method is to create sufficient KNN classifiers using different random subsets of selected features. This idea improves the accuracy, especially when there is a large number of features. Table 5 shows the accuracy results of intrusion detection using binary classification (natural and attack) and based on three different numbers of learners. Table 6 shows the accuracy results of RSL-KNN under multi-class classification (natural and different types of attacks) and based on three different numbers of learners. We can observe that RSL-KNN outperforms KNN under both classification tasks. As shown in Table 7, while the false positive rates of RSL-KNN under multi-class classification are between 0.3% and 0.4%, they are higher binary-class classification.

#### 6.1.2. Efficiency Evaluation

To evaluate the time cost of training and testing of RSL-KNN compared to KNN classifier, we train and test both classifiers on sub-dataset 9, which contains 5340 instances. This dataset is divided into 3738 instances for training and 1602 for testing and the time cost is measured for binary and multi-class classification. Table 8 shows the time of training and testing in seconds for both classifiers. We can notice that RSL-KNN incurs an insignificant additional time during the training. In the testing phase, RSL-KNN shows higher values than KNN.

### 6.2. Blockchain-Based Integrity Checking System

#### 6.2.1. Security Analysis

We analyze the security of BICS and discuss its resilience against the following attacks:

- *Unauthorized Access to SDN controller*: We assumed that the SDN controller is located in a private cloud, and only accessible from a single host. Thus, it is impossible for an external adversary to gain authorized access to the SDN controller. In addition, by applying an authentication and access control mechanism, we can prevent unauthorized hosts from accessing the network resources, as explained in [73]. Therefore, there is no way that fraudulent flow rules are generated from the SDN controller.
- *Man-in-the-middle attack between switch and controller*: As fraudulent flow rules cannot be generated from the SDN controller, and as the latter is the only node that has the right to create entries in the blockchain, therefore, the blockchain only stores legitimate flow rules. If the flow table of the vSwitch is poisoned with tampered rules, the firewall will eventually detect this attack after comparing the vSwitch logs and the rules stored in the blockchain.
- *vSwitch misconfiguration:* Like the Man-in-the-middle attack, if forged flow rules are injected in the flow table of the vSwitch, it is possible to detect this attack.
- *Flow table overflow attack*: External attackers can launch DoS/DDoS attack to inject a large number of flow rules, which leads to flow table overflow. All the injected flow rules will be detected by BICS after comparing them with ones stored in the blockchain, Hence, this attack can be mitigated by deleting each detected injected flow rule.
- *Blockchain poisoning:* Under this attack, an adversary impersonates an SDN controller and injects the same flow rule in both the vSwitch and the blockchain. This attack is not possible, as the blockchain is only updated by the SDN controller that has a unique private key.
- *Blockchain vulnerabilities* The blockchaim might suffer from many vulnerabilities such as:
  - 51% vulnerability: If a single miner has more than 50% of the total computing power of the blockchain, then it can hinder the normal operations of the blockchain.
  - Hiding blocks: The participant only exposes transactions that are in his favor.
  - Whitewhashing: The participant makes a new identity to get rid of his bad reputation)
  - Refusal to sign: The participant does not sign a transaction that is not in his favor.
  The above vulnerabilities cannot be exploited in case of BICS as we deploy a private blockchain, and the participants are trusted and are within the internal network. However, if the private key of the SDN controller is leaked, an adversary can exploit this vulnerability to launch some attacks such as tampering, impersonation, and Man-in-the-middle attack. Here, we consider two cases:
  - *External adversary:* If the adversary tries to use the private key to generate fake blocks, this attempt will be detected as the operation comes from outside the network, whereas the SDN controller is located inside the network.
  - *Internal adversary:* To prevent an internal adversary from using the private key and generate fake blocks, the SDN controller is only accessible from a single host and access control mechanism are implemented.
  In addition, to mitigate the risk of private key leakage, the network administrator needs to implement security controls related to key management.

#### 6.2.2. Performance Evaluation

We evaluate the performance of BICS by varying the number of false rules that are injected into the network. In order to perform this test, we disconnect the SDN controller and inject the rules at the switch-level. Table 9 summarizes the detection time and the detection rate of BICS. We observe that BICS achieves a detection rate of 100% with a very low detection time. The full detection rate is explained by the fact that the blockchain is immutable, i.e., it ensures that data once written to a blockchain cannot be altered. To ensure immutability, the blockchain is based on two main concepts: hashes and chains of blocks, which are proved mathematically to ensure data integrity. If an adversary creates a fraudulent flow rule and wants to inject it in the vSwitch, it cannot alter an existing flow rule in the blockchain and make it similar to a fraudulent one. In addition, as proved in Section 6.2.1, any injection of new forged flow rule in the flow table of the vSwitch is eventually detected. Moreover, it is important to mention that the detection time of BICS is scalable with respect to the number of injected rules.

To evaluate the execution time overhead of BICS, we measure the below metrics by varying the number of vSwitches that are deployed in the network.

- *Blockchain Creation Time (BCT):* is the time needed to create a blockchain block.
- *Log Retrieval Time (LRT):* is the time needed for the firewall to retrieve the switch log.
- *Rule Retrieval Time (RRT):* is the time needed for the firewall to retrieve the rules from the blockchain.
- *Processing Time (PT):* is the time needed for the firewall to compare the rules retrieved from blochain and the log retrieved from the switch.
- *Execution time overhead (ETO):* is the total execution time of the BICS operation, which is the sum of *BCT*, *LRT*, *RRT*, and *PT*.

Table 10 shows that *ETO* increases as the number of switches increases. This is because the firewall has to retrieve the log information from each switch, which affects *LRT*. On the other hand, we observe that *BCT*, *RRT*, and *PT* are low and are less affected by increasing the the number of switches. We can also observe that BICS incurs a very low block creation time compared to other public blockchain platforms, e.g., bitcoin, that requires around 10 min to create one block [74]. This is due to the fact that these blockchains run a consensus mechanism like Proof of Work (PoW) or Proof of State (PoS) in order to mine, validate and append a new block to the blockchain. In case of BICS, no consensus is required, and it is replaced with access rights that are assigned to known participants.

## 7. Conclusions

In this paper, we have proposed a security architecture for IoT-based industrial control systems, which integrates the Blockchain and the Software-defined wide-area network technologies. The proposed security architecture is composed of an intrusion detection system, named RSL-KNN, and a Blockchain-based Integrity Checking System (BICS). The proposed security solution has been tested on an Industrial Control System Cyber attack Dataset and on an experimental platform combining software-defined networking and blockchain technologies. The proposed security solution has produced an overall good performance. RSL-KNN has scored an accuracy of 96.73% and 91.07% under binary class and multi-class classification tasks, respectively. In addition, BICS can detect fraudulent flow rules at a detection rate of 100%, and is scalable in terms of detection time. As a part of future work, we plan to test more Industrial Control System (ICS) cyber attacks datasets, and apply different deep learning techniques for better IDS accuracy. Moreover, it would be interesting to leverage the blockchain technology to prevent injection of fraudulent flow rules in the flow tables, instead of only detecting them.

## Figures and Tables

**Table 1:** Distribution of datasets.

Parameter	Value
Sub-dataset 1	4966
Sub-dataset 2	5096
Sub-dataset 3	5415
Sub-dataset 4	5202
Sub-dataset 5	5161
Sub-dataset 6	4967
Sub-dataset 7	5236
Sub-dataset 8	5315
Sub-dataset 9	5340
Sub-dataset 10	5569
Sub-dataset 11	5251
Sub-dataset 12	5224
Sub-dataset 13	5271
Sub-dataset 14	5115
Sub-dataset 15	5276

**Table 2:** Parameters Settings of RSL method.

Parameter	Value
Batch Size	100
number Iterations	5, 15, 20
Seed	1
Subspace Size	0.5
Similarity function	Euclidean distance

**Table 3:** Accuracy results under binary classification.

Dataset	LSVM	BN	NB-K	KNN	AdaBoostM1	Bagging	DT	RF
Sub-dataset 1	78.27%	73.42%	71.93%	94.38%	93.60%	92.87%	92.65%	95.53%
Sub-dataset 3	71.39%	75.55%	68.50%	94.40%	92.95%	91.86%	92.41%	94.92%
Sub-dataset 5	71.61%	73.86%	71.13%	95.16%	93.41%	91.65%	92.81%	95.47%
Sub-dataset 7	69.46%	71.80%	58.88%	93.50%	91.87%	89.98%	88.60%	93.33%
Sub-dataset 9	69.46%	71.80%	58.88%	93.50%	91.87%	89.98%	88.60%	93.33%
Sub-dataset 10	70.86%	77.93%	71.23%	94.65%	92.78%	91.76%	92.93%	95.44%
Sub-dataset 13	77.94%	75.34%	75.74%	95.49%	94.10%	92.20%	92.91%	95.96%
Sub-dataset 15	64.69%	73.16%	68.33%	93.33%	92.53%	91.41%	90.35%	94.81%

**Table 4:** Accuracy results under multi-class classification.

Dataset	LSVM	BN	NB-K	KNN	AdaBoostM1	Bagging	DT	RF
Sub-dataset 1	27.51%	71.65%	16.17%	86.25%	25.29%	79.56%	9.01%	79.21%
Sub-dataset 3	28.99%	73.11%	18.76%	87.66%	22.53%	77.40%	8.46%	77.51%
Sub-dataset 5	27.17%	74.64%	19.71%	87.04%	21.00%	79.15%	9.01%	78.78%
Sub-dataset 7	29.79%	73.70%	14.32%	86.90%	20.61%	78.61%	74.57%	77.85%
Sub-dataset 9	30.36%	70.21%	18.61%	85.86%	25.02%	74.23%	25.60%	74.08%
Sub-dataset 10	23.56%	73.30%	17.01%	87.59%	19.38%	79.24%	24.74%	78.54%
Sub-dataset 13	27.43%	72.89%	13.79%	87.00%	19.35%	75.79%	82.60%	78.62%
Sub-dataset 15	27.54%	73.88%	19.45%	87.06%	29.44%	72.37%	83.19%	73.29%

**Table 5:** Accuracy results of Random Subspace Learning-based K-Nearest Neighbor (RSL-KNN) under binary classification.

Dataset	5	15	20
Sub-dataset 1	95.7108%	95.8921%	95.8719%
Sub-dataset 2	95.0483%	95.2456%	95.1272%
Sub-dataset 3	95.7341%	95.9926%	95.9187%
Sub-dataset 4	95.0980%	95.1749%	95.1749%
Sub-dataset 5	96.2798%	96.3767%	96.5511%
Sub-dataset 6	95.0473%	95.7318%	95.7318%
Sub-dataset 7	94.9007%	95.3209%	95.2636%
Sub-dataset 8	95.0517%	95.3904%	95.5974%
Sub-dataset 9	94.7753%	95.1685%	95.1685%
Sub-dataset 10	95.5827%	96.0855%	96.1393%
Sub-dataset 11	95.7913%	95.9246%	95.7722%
Sub-dataset 12	95.5781%	95.7695%	95.9418%
Sub-dataset 13	96.2626%	96.7179%	96.7369%
Sub-dataset 14	95.3275%	95.4839%	95.4839%
Sub-dataset 15	94.8635%	95.0152%	95.1099%

**Table 6:** Accuracy results of RSL-KNN under multi-class classification.

Dataset	5	15	20
Sub-dataset 1	89.3274%	89.7704%	89.9919%
Sub-dataset 2	88.3212%	88.9130 %	89.1300%
Sub-dataset 3	90.2678%	90.5817%	90.7295%
Sub-dataset 4	89.2349%	89.3118%	89.7924%
Sub-dataset 5	89.9632%	90.2538%	90.2151%
Sub-dataset 6	89.7725%	90.6986%	90.7389%
Sub-dataset 7	89.5913%	89.6677%	89.8778%
Sub-dataset 8	89.8024%	90.5550 %	90.6115%
Sub-dataset 9	88.6891%	88.9139%	88.8015%
Sub-dataset 10	90.1239%	90.7883%	90.5189%
Sub-dataset 11	89.6210%	90.2495%	90.3828%
Sub-dataset 12	90.1417%	91.0796%	91.0605%
Sub-dataset 13	90.1347%	90.5141%	90.4003%
Sub-dataset 14	88.6413%	89.1300%	89.4233%
Sub-dataset 15	89.2911%	89.9545%	90.0872%

**Table 7:** False positive rate of RSL-KNN under binary and multi-class classification.

	Binary-Class Classification			Multi-Class Classification		
Dataset	5	15	20	5	15	20
Sub-dataset 1	8.4%	8.3%	8.5%	0.4%	0.3%	0.3%
Sub-dataset 2	7.2%	7.0%	7.0%	0.4%	0.4%	0.3%
Sub-dataset 3	6.3%	6.3%	6.3%	0.3%	0.3%	0.3%
Sub-dataset 4	6.4%	6.4%	6.4%	0.4%	0.4%	0.4%
Sub-dataset 5	5.7%	5.8%	5.6%	0.3%	0.3%	0.3%
Sub-dataset 6	7.1%	6.3%	6.1%	0.3%	0.3%	0.3%
Sub-dataset 7	8.5%	8.4%	8.3%	0.3%	0.3%	0.3%
Sub-dataset 8	7.4%	7.1%	6.7%	0.3%	0.3%	0.3%
Sub-dataset 9	6.9%	6.6%	6.6%	0.4%	0.4%	0.4%
Sub-dataset 10	6.4%	5.5%	5.5%	0.4%	0.3%	0.4%
Sub-dataset 11	7.9%	7.8%	8.0%	0.3%	0.3%	0.3%
Sub-dataset 12	5.6%	6.7%	5.3%	0.3%	0.3%	0.3%
Sub-dataset 13	7.7%	7.2%	7.0%	0.4%	0.4%	0.4%
Sub-dataset 14	7.7%	7.5%	7.4%	0.4%	0.4%	0.4%
Sub-dataset 15	6.2%	6.1%	5.8%	0.4%	0.3%	0.3%

**Table 8:** Efficiency results of RSL-KNN and KNN.

Classifier Model	Training Time on 3738 Instances	Testing Time on 1602 Instances	Task
KNN	0.01	0.88	Binary class classification
	0.01	6.45	Multi-class classification
RSL-KNN	0.05	3.64	Binary class classification
	0.05	15.04	Multi-class classification

**Table 9:** Detection performance of BICS.

Number of Injected Rules	Detection Time (s)	Detection Rate (%)
10	2.40	100%
50	2.43	100%
100	2.44	100%
200	2.45	100%
500	2.54	100%
750	2.57	100%
1000	2.64	100%
1500	2.70	100%
2000	2.83	100%

**Table 10:** Execution time overhead of BICS.

Number of vSwitches	BCT (s)	LRT (s)	RRT (s)	PT (s)	ETO (s)
10	0.248	2.339	0.143	0.003	2.485
20	0.336	2.592	0.176	0.008	2.77
30	0.194	2.876	0.202	0.018	3.097
40	0.190	3.059	0.146	0.028	3.233
50	0.228	3.555	0.160	0.043	3.759
60	0.311	3.721	0.169	0.064	3.954
70	0.324	4.246	0.170	0.105	4.522
80	0.333	4.544	0.155	0.154	4.854
90	0.412	5.400	0.228	0.146	5.774
100	0.443	5.724	0.238	0.191	6.154

## References

[1] Da Xu L., He W., Li S. (2014). Internet of things in industries: A survey. IEEE Trans. Ind. Inform..

[2] Zuehlke D. (2010). Smart Factory—Towards a Factory-of-Things. ELSEVIER Annu. Rev. Control.

[3] Hasan M.M., Mouftah H.T. (2016). Optimal trust system placement in smart grid SCADA networks. IEEE Access.

[4] Sajid A., Abbas H., Saleem K. (2016). Cloud-assisted IoT-based SCADA systems security: A review of the state of the art and future challenges. IEEE Access.

[5] Shahzad A., Kim Y.G., Elgamoudi A. Secure IoT platform for industrial control systems. Proceedings of the International Conference on Platform Technology and Service (PlatCon).

[6] ENISA Communication Network Dependencies for ICS/SCADA Systems. https://www.enisa.europa.eu/publications/ics-scada-dependencies.

[7] Polityuk P., Vukmanovic O., Jewkes S. (2017). Ukraine’s Power Outage Was a Cyber Attack: Ukrenergo.

[8] Farhady H., Lee H., Nakao A. (2015). Software-defined networking: A survey. Comput. Netw..

[9] Nunes B.A.A., Mendonca M., Nguyen X.N., Obraczka K., Turletti T. (2014). A survey of software-defined networking: Past, present, and future of programmable networks. IEEE Commun. Surv. Tutor..

[10] Michel O., Keller E. SDN in wide-area networks: A survey. Proceedings of the Fourth International Conference on Software Defined Systems (SDS).

[11] Gartner (2015). Predicting SD-WAN Adoption. https://blogs.gartner.com/andrew-lerner/2015/12/15/predicting-sd-wan-adoption/.

[12] Al-Shaer E., Al-Haj S. FlowChecker: Configuration analysis and verification of federated OpenFlow infrastructures. Proceedings of the 3rd ACM Workshop on Assurable and Usable Security Configuration.

[13] Porras P., Shin S., Yegneswaran V., Fong M., Tyson M., Gu G. A security enforcement kernel for OpenFlow networks. Proceedings of the First Workshop on Hot Topics in Software Defined Networks.

[14] Khurshid A., Zou X., Zhou W., Caesar M., Godfrey P.B. Veriflow: Verifying network-wide invariants in real time. Presented as Part of the 10th USENIX Symposium on Networked Systems Design and Implementation (NSDI).

[15] Adhikari U., Pan S., Morris T., Borges R., Beave J. Industrial Control System (ICS) Cyber Attack Datasets. https://sites.google.com/a/uah.edu/tommy-morris-uah/ics-data-sets.

[16] Hink R.C.B., Beaver J.M., Buckner M.A., Morris T., Adhikari U., Pan S. Machine learning for power system disturbance and cyber-attack discrimination. Proceedings of the 7th International Symposium on Resilient Control Systems (ISRCS).

[17] Maglaras L.A., Kim K.H., Janicke H., Ferrag M.A., Rallis S., Fragkou P., Maglaras A., Cruz T.J. (2018). Cyber security of critical infrastructures. ICT Express.

[18] Maglaras L.A., Jiang J. Intrusion detection in SCADA systems using machine learning techniques. Proceedings of the Science and Information Conference (SAI).

[19] Hu Y., Yang A., Li H., Sun Y., Sun L. (2018). A survey of intrusion detection on industrial control systems. Int. J. Distrib. Sens. Netw..

[20] Abokifa A.A., Haddad K., Lo C., Biswas P. (2018). Real-Time Identification of Cyber-Physical Attacks on Water Distribution Systems via Machine Learning–Based Anomaly Detection Techniques. J. Water Resour. Plan. Manag..

[21] Narayanan V., Bobba R.B. Learning Based Anomaly Detection for Industrial Arm Applications. Proceedings of the 2018 Workshop on Cyber-Physical Systems Security and PrivaCy.

[22] Caselli M., Zambon E., Kargl F. Sequence-aware intrusion detection in industrial control systems. Proceedings of the 1st ACM Workshop on Cyber-Physical System Security.

[23] Khalili A., Sami A., Khozaei A., Pouresmaeeli S. (2018). SIDS: State-based intrusion detection for stage-based cyber physical systems. Int. J. Crit. Infrastruct. Prot..

[24] Zhang F., Kodituwakku H.A.D.E., Hines W., Coble J.B. (2019). Multi-Layer Data-Driven Cyber-Attack Detection System for Industrial Control Systems Based on Network, System and Process Data. IEEE Trans. Ind. Inform..

[25] Ghaeini H.R., Antonioli D., Brasser F., Sadeghi A.R., Tippenhauer N.O. State-aware anomaly detection for industrial control systems. Proceedings of the 33rd Annual ACM Symposium on Applied Computing.

[26] Wang W., Xie Y., Ren L., Zhu X., Chang R., Yin Q. Detection of data injection attack in industrial control system using long short term memory recurrent neural network. Proceedings of the 13th IEEE Conference on Industrial Electronics and Applications (ICIEA).

[27] Li D., Guo H., Zhou J., Zhou L., Wong J.W. (2019). SCADAWall: A CPI-enabled firewall model for SCADA security. Comput. Secur..

[28] Serpanos D. (2018). Secure and Resilient Industrial Control Systems. IEEE Des. Test.

[29] Serpanos D., Khan M.T., Shrobe H. (2018). Designing Safe and Secure Industrial Control Systems: A Tutorial Review. IEEE Des. Test.

[30] Falco G., Caldera C., Shrobe H. (2018). IIOT cybersecurity risk modeling for scada systems. IEEE Internet Things J..

[31] Wood A., He Y., Maglaras L., Janicke H. (2017). A security architectural pattern for risk management of industry control systems within critical national infrastructure. Int. J. Crit. Infrastruct..

[32] Cook A., Smith R., Maglaras L., Janicke H. Measuring the risk of cyber attack in industrial control systems. Proceedings of the 4th International Symposium for ICS & SCADA Cyber Security Research 2016 (ICS-CSR 2016).

[33] Nourian A., Madnick S. (2018). A systems theoretic approach to the security threats in cyber physical systems applied to stuxnet. IEEE Trans. Dependable Secur. Comput..

[34] Nasr P.M., Yazdian-Varjani A. (2018). Toward Operator Access Management in SCADA System: Deontological Threat Mitigation. IEEE Trans. Ind. Inform..

[35] Ferrag M.A., Derdour M., Mukherjee M., Derhab A., Maglaras L., Janicke H. (2019). Blockchain Technologies for the Internet of Things: Research Issues and Challenges. IEEE Internet Things J..

[36] Košt’ál K., Helebrandt P., Belluš M., Ries M., Kotuliak I. (2019). Management and Monitoring of IoT Devices Using Blockchain. Sensors.

[37] Agyekum O., Opuni-Boachie K., Xia Q., Sifah E.B., Gao J., Xia H., Du X., Guizani M. (2019). A Secured Proxy-Based Data Sharing Module in IoT Environments Using Blockchain. Sensors.

[38] Yang J., He S., Xu Y., Chen L., Ren J. (2019). A Trusted Routing Scheme Using Blockchain and Reinforcement Learning for Wireless Sensor Networks. Sensors.

[39] Liang G., Weller S.R., Luo F., Zhao J., Dong Z.Y. (2018). Distributed blockchain-based data protection framework for modern power systems against cyber attacks. IEEE Trans. Smart Grid.

[40] Aitzhan N.Z., Svetinovic D. (2018). Security and privacy in decentralized energy trading through multi-signatures, blockchain and anonymous messaging streams. IEEE Trans. Dependable Secur. Comput..

[41] Cahn A., Hoyos J., Hulse M., Keller E. Software-defined energy communication networks: From substation automation to future smart grids. Proceedings of the IEEE International Conference on Smart Grid Communications (SmartGridComm).

[42] da Silva E.G., Knob L.A.D., Wickboldt J.A., Gaspary L.P., Granville L.Z., Schaeffer-Filho A. Capitalizing on SDN-based SCADA systems: An anti-eavesdropping case-study. Proceedings of the IFIP/IEEE International Symposium on Integrated Network Management (IM).

[43] Aydeger A., Akkaya K., Uluagac A.S. SDN-based resilience for smart grid communications. Proceedings of the IEEE Conference on Network Function Virtualization and Software Defined Network (NFV-SDN).

[44] Zhang X., Wei K., Guo L., Hou W., Wu J. SDN-based resilience solutions for smart grids. Proceedings of the International Conference on Software Networking (ICSN).

[45] Aydeger A., Akkaya K., Cintuglu M.H., Uluagac A.S., Mohammed O. Software defined networking for resilient communications in smart grid active distribution networks. Proceedings of the IEEE International Conference on Communications (ICC).

[46] Ren L., Qin Y., Wang B., Zhang P., Luh P.B., Jin R. (2017). Enabling resilient microgrid through programmable network. IEEE Trans. Smart Grid.

[47] Al-Rubaye S., Kadhum E., Ni Q., Anpalagan A. (2019). Industrial internet of things driven by SDN platform for smart grid resiliency. IEEE Internet Things J..

[48] Da Silva E.G., da Silva A.S., Wickboldt J.A., Smith P., Granville L.Z., Schaeffer-Filho A. A One-Class NIDS for SDN-Based SCADA Systems. Proceedings of the IEEE 40th Annual Computer Software and Applications Conference (COMPSAC).

[49] Ghosh U., Chatterjee P., Shetty S. A Security Framework for SDN-Enabled Smart Power Grids. Proceedings of the IEEE 37th International Conference on Distributed Computing Systems Workshops (ICDCSW).

[50] Dargahi T., Caponi A., Ambrosin M., Bianchi G., Conti M. (2017). A survey on the security of stateful SDN data planes. IEEE Commun. Surv. Tutor..

[51] Kreutz D., Ramos F.M., Verissimo P., Rothenberg C.E., Azodolmolky S., Uhlig S. (2015). Software-defined networking: A comprehensive survey. Proc. IEEE.

[52] Li H., Li P., Guo S., Nayak A. (2014). Byzantine-resilient secure software-defined networks with multiple controllers in cloud. IEEE Trans. Cloud Comput..

[53] Mohan P.M., Truong-Huu T., Gurusamy M. Primary-backup controller mapping for Byzantine fault tolerance in software defined networks. Proceedings of the IEEE Global Communications Conference (GLOBECOM 2017).

[54] Sridharan V., Gurusamy M. Game-Theoretic Approach to Malicious Controller Detection in Software Defined Networks. Proceedings of the IEEE International Conference on Communications (ICC).

[55] What is OpenFlow? Definition and How it Relates to SDN. https://www.sdxcentral.com/sdn/definitions/what-is-openflow/.

[56] Pavel Cheremushkin S.T. (2018). OPC UA Security Analysis. https://securelist.com/opc-ua-security-analysis/85424/.

[57] Dreier J., Puys M., Potet M.L., Lafourcade P., Roch J.L. Formally verifying flow integrity properties in industrial systems. Proceedings of the 14th International Conference on Security and Cryptography (SECRYPT 2017).

[58] Puys M., Potet M.L., Lafourcade P. Formal analysis of security properties on the OPC-UA SCADA protocol. Proceedings of the International Conference on Computer Safety, Reliability, and Security.

[59] Amoah R. (2016). Formal Security Analysis of the DNP3-Secure Authentication Protocol. Ph.D. Thesis.

[60] Barandiaran I. (1998). The random subspace method for constructing decision forests. IEEE Trans. Pattern Anal. Mach. Intell..

[61] Bryll R., Gutierrez-Osuna R., Quek F. (2003). Attribute bagging: Improving accuracy of classifier ensembles by using random feature subsets. Pattern Recognit..

[62] Tao D., Tang X., Li X., Wu X. (2006). Asymmetric bagging and random subspace for support vector machines-based relevance feedback in image retrieval. IEEE Trans. Pattern Anal. Mach. Intell..

[63] Bertoni A., Folgieri R., Valentini G. (2005). Bio-molecular cancer prediction with random subspace ensembles of support vector machines. Neurocomputing.

[64] Skurichina M., Duin R.P. (2002). Bagging, boosting and the random subspace method for linear classifiers. Pattern Anal. Appl..

[65] Hosseini M.P., Hajisami A., Pompili D. Real-time epileptic seizure detection from EEG signals via random subspace ensemble learning. Proceedings of the IEEE International Conference on Autonomic Computing (ICAC).

[66] Witten I., Frank E. Data Mining Software in Java. http://www.cs.waikato.ac.nz/ml/weka.

[67] Openstack. https://www.openstack.org/.

[68] MultiChain. https://www.multichain.com/.

[69] Bitcoin Core. https://bitcoin.org/en/bitcoin-core/.

[70] JSON-RPC 2.0 Specification. https://www.jsonrpc.org/specification.

[71] Onos. https://www.opennetworking.org/onos/.

[72] Mininet. http://mininet.org/.

[73] Mattos D.M.F., Duarte O.C.M.B. (2016). AuthFlow: Authentication and access control mechanism for software defined networking. Ann. Telecommun..

[74] Average Time to Mine a Block in Minutes. https://data.bitcoinity.org/bitcoin/block_time/5y?f=m10&t=l.

